# Supplementary material for: Cytokine and T cell responses in post-chikungunya viral arthritis: A cross-sectional study
Source: PLoS One. 2024 Mar 20;19(3):e0299521. doi: 10.1371/journal.pone.0299521 (PMC10954186; doi:10.1371/journal.pone.0299521)
Supplement: Attachment — Submitted filename: Response to Reviewers.docx [file pone.0299521.s002.docx]

PONE-D-23-30658

Cytokine and T cell responses in post-chikungunya viral arthritis: A cross-sectional study

PLOS ONE

**Response to Reviewers:**

**Academic Review:**

Please simplify some part of the presentation and carefully defined the arthritis flare as an example.

Thank you for your review. We have unified the participant recruitment description into the study design section as suggested. We have clearly defined arthritis flare and removed references to arthritis relapses as suggested.

**Journal requirements:**

When submitting your revision, we need you to address these additional requirements.

1. Please ensure that your manuscript meets PLOS ONE's style requirements, including those for file naming. The PLOS ONE style templates can be found at

https://journals.plos.org/plosone/s/file?id=wjVg/PLOSOne_formatting_sample_main_body.pdf and

<https://journals.plos.org/plosone/s/file?id=ba62/PLOSOne_formatting_sample_title_authors_affiliations.pdf>

Thank you, we confirm adherence to the PLOS ONE style requirement.

2. Note from Emily Chenette, Editor in Chief of PLOS ONE, and Iain Hrynaszkiewicz, Director of Open Research Solutions at PLOS: Did you know that depositing data in a repository is associated with up to a 25% citation advantage (https://doi.org/10.1371/journal.pone.0230416)? If you’ve not already done so, consider depositing your raw data in a repository to ensure your work is read, appreciated and cited by the largest possible audience. You’ll also earn an Accessible Data icon on your published paper if you deposit your data in any participating repository (<https://plos.org/open-science/open-data/#accessible-data>).

Our de-identified trial dataset is limited to a single excel that we will upload as a supplementary file for open access.

3. Thank you for stating the following in the Competing Interests section:

“I have read the journal's policy and the authors of this manuscript have the following competing interests: AYC reports consulting for Valneva. RLA reports stock ownership of Abbvie, Pfizer, Johnson & Johnson, & Briston Myers Squibb. GSF reports consulting for Eli Lilly. Other authors report no commercial interest or conflicts of interest in relation to this work.”

Please confirm that this does not alter your adherence to all PLOS ONE policies on sharing data and materials, by including the following statement: "This does not alter our adherence to PLOS ONE policies on sharing data and materials.” (as detailed online in our guide for authors http://journals.plos.org/plosone/s/competing-interests).  If there are restrictions on sharing of data and/or materials, please state these. Please note that we cannot proceed with consideration of your article until this information has been declared.

Please include your updated Competing Interests statement in your cover letter; we will change the online submission form on your behalf.

We confirm this statement and have added this to our cover letter, “This does not alter our adherence to PLOS ONE policies on sharing data and materials”.

4. Please include captions for your Supporting Information files at the end of your manuscript, and update any in-text citations to match accordingly. Please see our Supporting Information guidelines for more information: <http://journals.plos.org/plosone/s/supporting-information>.

We have added a caption while adding the dataset as a supplementary file as per the naming guidelines provided.

5. Please review your reference list to ensure that it is complete and correct. If you have cited papers that have been retracted, please include the rationale for doing so in the manuscript text, or remove these references and replace them with relevant current references. Any changes to the reference list should be mentioned in the rebuttal letter that accompanies your revised manuscript. If you need to cite a retracted article, indicate the article’s retracted status in the References list and also include a citation and full reference for the retraction notice.

The references have been reviewed.

**Reviewer #1:** The manuscript: “Cytokine and T cell responses in post-chikungunya viral arthritis: A cross-sectional study”. The main objective of this study was “to define the relationship between chronic chikungunya post-viral arthritis disease severity, cytokine response and T cell subsets in order to identify potential targets for therapy”, for which were included participants with chikungunya arthritis.

The methods used such as type of study, study population and the questionaries used for the evaluation of the severity were adequate. The data, the analysis and interpreted of the these were accurately and adequate for addressing the research question, also were enough to draw conclusions. The results are presented in adequate, and the discussion was concrete and well supported.

The authors provided enough information to validate the study.

The study has a limitation with relation to that is not included healthy control, however, the results obtained comparing different groups according to the severity of the disease drive valid results.
I think that it is well.

Other observations

In the “Participants”, “Study Design" and “Statistical analyses” sections, the authors described the study population ("participants"). On the other hand, in “Statistical analyses” section, was describe the way in which they were recruited.
I suggest that the different descriptions of the participants and the way of recruitment be included in a single section. This adjustment would allow unify this concept in one single section.

In the section: “Statistical analyses”, the authors included the study design.
I suggest, if the authors are agreed, that the study design be included in the section “Study Design".

Thank you for this suggestion, the all the participant recruitment information was moved into the ‘Study Design’ section.

I suggest, if it is possible, describe the sensibility and specificity of IgG antibody immunofluorescence (EUROIMMUN) test.

This information has been added to the ‘Chikungunya IgG Immunofluorescence’ section, “This assay has a sensitivity of 97% and specificity of 96%.”

In row 333 the word "also" is not appropriate with the finding

Thank you, ‘also’ has been removed.

In row 343 the following phrase: “the deficiency of which promotes Treg accumulation in inflamed issue(17).”, could be more appropriate for the discussion..

This comment has been moved to the discussion.

In the text and in the tables is used the concepts, "Arthritis-Flare" and "arthritis relapse". I suggest that be used only single of these, for example "Arthritis-Flare".

In the ‘Disease Activity’ section, the definition of an arthritis flare is further elaborated and all references to ‘relapses’ has been changed to ‘flares’ for improved clarity.
