# Supplementary material for: Cytokine and T cell responses in post-chikungunya viral arthritis: A cross-sectional study
Source: PLoS One. 2024 Mar 20;19(3):e0299521. doi: 10.1371/journal.pone.0299521 (PMC10954186; doi:10.1371/journal.pone.0299521)
Supplement: Attachment — Submitted filename: Response to Reviewers.docx [file pone.0299521.s003.docx]

PONE-D-23-30658

Cytokine and T cell responses in post-chikungunya viral arthritis: A cross-sectional study

PLOS ONE

**Response to Reviewers:**

**Academic Review:**

As noted there is some mistake in the cohort description as a patient below or from 18 years of age were seems to have been included in the study. Please check if discarding the data from these patients (specifically the youngest one) impact or not the statistical analysis. Please provide a new computation at least for the reviewer. If there are not modification discard these patients that are not in the described cohort and refresh all the results. If there is a modification please provide some explanation and indicate this as a major limitation of your study.

Thank you very much for catching this error. I have checked our source documents and participant 75 is not 6 years of age but rather 59 years of age. There was a typo when transcribing from the source documents and the age was confirmed with their date of birth from their identification card. This change has been made in the dataset and the mean ages edited accordingly in Table 1. Furthermore, the text at row 117 has been edited to reflect that participants were age 18 and older in this study.

**Reviewer #1:**

1. The authors made the changes suggested in the first evaluation. However, I have a new comment that is important to clarify before publishing the manuscript:

1. In the row 117 describes the following: “Participants over 18 years of age with a history of CHIKV infection were analyzed,” but the database includes one 6-year-old participant and three 18-year-old participants. I suggest two options: 1) that this data be clarified in the manuscript, for example "Participants 18 years of age or older and one participant 6 years of age with a history of CHIKV infection were analyzed" or 2) that the age data be removed in this row, for example: "Participants with a history of CHIKV infection were analyzed", because the mean and standard deviation of the age of the participants are described in the results.

Thank you very much. Please see the response from above.
